# Ethyl 3-(2-chloro-5,8-dimeth­oxy­quinolin-3-yl)-2-cyano­oxirane-2-carboxyl­ate

**DOI:** 10.1107/S1600536811023336

**Published:** 2011-06-22

**Authors:** Hayette Alliouche, Sofiane Bouacida, Thierry Roisnel, Ali Belfaitah

**Affiliations:** a Laboratoire des Produits Naturels d’Origine, Végétale et de Synthèse Organique, PHYSYNOR, Université Mentouri-Constantine, 25000 Constantine, Algeria; b Unité de Recherche de Chimie de l’Environnement et Moléculaire Structurale, CHEMS, Université Mentouri-Constantine, 25000 Algeria; c Centre de Difractométrie X, UMR 6226 CNRS Unité Sciences Chimiques de Rennes, Université de Rennes I, 263 Avenue du Général Leclerc, 35042 Rennes, France

## Abstract

The title mol­ecule, C_17_H_15_ClN_2_O_5_, contains a quinolyl unit linked to a functionalized oxirane system with a 2,3-*trans* arrangement of the substituents (ester group *versus* quinol­yl). The structure can be described as being built up from zigzag layers parallel to (1
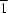
0). The heterocyclic ring of the quinolyl unit forms a dihedral angle of 60.05 (1)° with the oxirane plane. The crystal packing is stabilized by inter­molecular C—H⋯O and C—H⋯N hydrogen bonding, resulting in the formation of an infinite three-dimensional network and reinforcing the cohesion between the layers.

## Related literature

For applications of quinoline derivatives, see: Kansagra *et al.* (2000 ▶); Vasquez *et al.* (2004 ▶); Guo *et al.* (2009 ▶) Cunico *et al.* (2006 ▶); Mahamoud *et al.* (2006 ▶); Kumar *et al.* (2008 ▶); Hong *et al.* (2010 ▶).  For the biological activity of naturally occurring oxiranes, see: Bino (1980 ▶); Cross (1960 ▶); Marco-Contelles *et al.* (2004 ▶); Pearson & Ong (1981 ▶). For applications of oxiranes, see: Hanson (1991 ▶); Kumar & Leelavathi (2007 ▶); Das *et al.* (2007 ▶); Boukhris *et al.* (1996 ▶); Ammadi *et al.*, (1999 ▶). For our previous work on the preparation of quinoline derivatives, see: Bouraiou *et al.* (2008 ▶); Benzerka *et al.* (2008 ▶); Ladraa *et al.* (2010 ▶). For weak hydrogen bonds, see: Desiraju & Steiner, (1999 ▶).
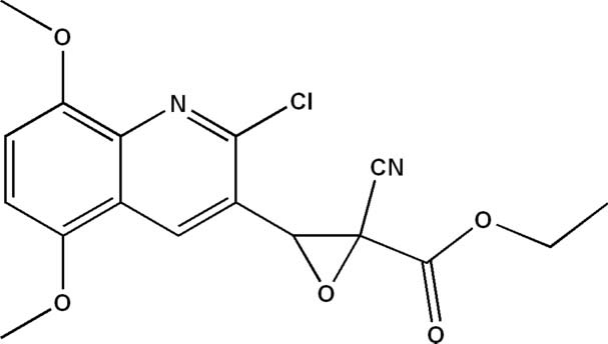


## Experimental

### 

#### Crystal data

C_17_H_15_ClN_2_O_5_
*M* *_r_* = 362.76
Triclinic, 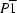

*a* = 8.3784 (3) Å
*b* = 10.1071 (4) Å
*c* = 10.7027 (4) Å
α = 102.489 (2)°
β = 103.977 (2)°
γ = 96.026 (2)°
*V* = 846.77 (6) Å^3^
*Z* = 2
Mo *K*α radiation
μ = 0.26 mm^−1^
*T* = 150 K
0.28 × 0.21 × 0.12 mm

#### Data collection

Bruker APEXII diffractometer
Absorption correction: multi-scan (*SADABS*; Sheldrick, 2002 ▶) *T*_min_ = 0.842, *T*_max_ = 0.970
12867 measured reflections
3803 independent reflections
3369 reflections with *I* > 2σ(*I*)
*R*_int_ = 0.020

#### Refinement

*R*[*F* ^2^ > 2σ(*F* ^2^)] = 0.049
*wR*(*F* ^2^) = 0.141
*S* = 1.02
3803 reflections
229 parameters
H-atom parameters constrained
Δρ_max_ = 0.54 e Å^−3^
Δρ_min_ = −0.42 e Å^−3^

### 

Data collection: *APEX2* (Bruker, 2009 ▶); cell refinement: *SAINT* (Bruker, 2009 ▶); data reduction: *SAINT*; program(s) used to solve structure: *SIR2002* (Burla *et al.*, 2005 ▶); program(s) used to refine structure: *SHELXL97* (Sheldrick, 2008 ▶); molecular graphics: *ORTEP-3 for Windows* (Farrugia, 1997 ▶) and *DIAMOND* (Brandenburg & Berndt, 2001 ▶); software used to prepare material for publication: *WinGX* (Farrugia, 1999 ▶).

## Supplementary Material

Crystal structure: contains datablock(s) global, I. DOI: 10.1107/S1600536811023336/zj2015sup1.cif

Structure factors: contains datablock(s) I. DOI: 10.1107/S1600536811023336/zj2015Isup2.hkl

Supplementary material file. DOI: 10.1107/S1600536811023336/zj2015Isup3.cml

Additional supplementary materials:  crystallographic information; 3D view; checkCIF report

## Figures and Tables

**Table 1:** Hydrogen-bond geometry (Å, °)

*D*—H⋯*A*	*D*—H	H⋯*A*	*D*⋯*A*	*D*—H⋯*A*
C7—H7⋯N19^i^	0.95	2.57	3.434 (3)	151
C24—H24*A*⋯O16^ii^	0.98	2.57	3.152 (4)	118

Symmetry codes: (i) 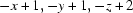; (ii) 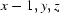.

**Fig. 1.**
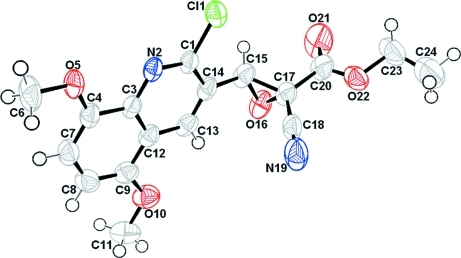
(Farrugia, 1997) the structure of the title compound with the atomic labelling scheme.Displacement are drawn at the 50% probability level.

**Fig. 2.**
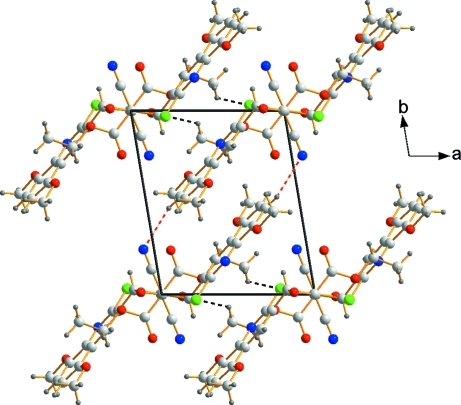
(Brandenburg & Berndt, 2001) A diagram of the layered crystal packing of (I) viewed down the b axis and showing hydrogen bond [N—H···O and C—H···O] as dashed line.

**Fig. 3.**
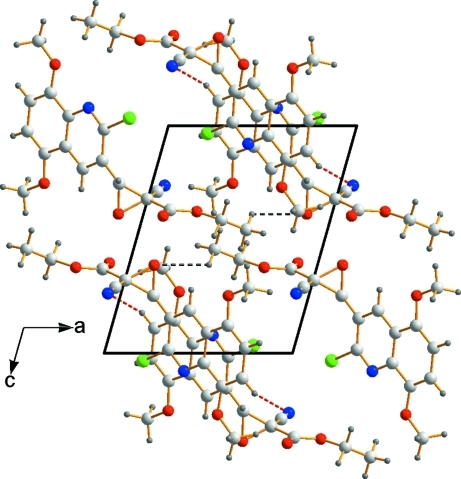
(Brandenburg & Berndt, 2001)A packing diagram of (I)viewed down the b axis.

## supplementary crystallographic information

### Comment

Due to their presence in a large number of natural products and bioactive compounds and their close association with the biological activities, quinoline and their derivatives have been extensively investigated by organic and biological chemists (Kansagra *et al.*, 2000; Vasquez *et al.*, 2004; Guo *et al.*, 2009). They are used in production of anti-malarial, antibiotics, anti-hypertension, anti-diabetic and so many other drugs (Cunico *et al.*, 2006; Mahamoud *et al.*, 2006; Kumar *et al.* 2008; Hong *et al.* 2010).

Oxiranes are important intermediates and starting materials which have found much use in synthetic organic chemistry owing to their ease of formation and ready activity toward nucleophiles (Hanson *et al.*, 1991; Kumar *et al.*, 2007; Das *et al.* 2007). In addition, natural occurring oxiranes are associated with various biological activities (Cross, 1960; Bino, 1980; Pearson *et al.*, 1981; Marco-Contelles *et al.*, 2004). 2-cyano-2-alkoxycarbonyloxiranes proved to be versatile reagents from which a large variety of compounds might be synthesized (Boukhris *et al.*, 1996; Ammadi *et al.*, 1999). In connection with our research program aimed at the synthesis and the biological evaluation of quinoline derivatives (Bouraiou *et al.*, 2008; Benzerka *et al.*, 2008; Ladraa *et al.*, 2010), we report in this paper the synthesis and the structure determination by X-ray of a new quinoline compound where quinolyl moiety is linked to functionalized oxirane system. The reactivity of this compound and its analogues toward nucleophiles is under investigation.

The molecular geometry and the atom-numbering scheme of (I) are shown in Fig. 1.

In the asymmetric unit of title compound the oxiranes unit bearing an ester and cyano groups at C3 and quinolyl moiety at C2.

The two rings of quinolyl moiety are fused in an axial fashion and form a dihedral angle of 2.43 (5)°. The heterocycle ring of quinolyl unit form also with oxirane plane a dihedral angle of 60.05 (1)°.

The crystal packing can be described as layers in zig zag parallel to (1–10) plane (Fig. 2). A weak hydrogen bond interactions (C—H···N=3.434 (3) Å)along the [110] directions ensure the stability in the same layer. (as reported by Desiraju & Steiner, 1999) These layers are linked together by a classical weak C—H···O interactions and π-π stacking The crystal packing is stabilized by intra and intermolecular hydrogen bond (C—H···N and C—H···O) and π-π stacking, resulting in the formation of infinite three-dimensional network linked these layers toghter and reinforcing a cohesion of structure (Fig. 3). Hydrogen-bonding parameters are listed in table 1.

### Experimental

The title compound was obtained by oxidation of (*E*)-ethyl-3-(2-chloro-5,8-dimethoxyquinolin-3-yl)-2-cyanoacrylate with 2,5 equivalents of *m*-chloroperoxybenzoic acid in dichloromethane at room temperature in the presence of 1,2 equivalents of potassium carbonate. Column chromatography (silica gel, eluant: CH_2_Cl_2_) of the residue afforded pure product as yellow solid. Crystals suitable for X-ray analysis were obtained by slow evaporation of a dichloromethane / methanol solution.

### Refinement

All non-H atoms were refined with anisotropic atomic displacement parameters. All H atoms were localized on Fourier maps but introduced in calculated positions and treated as riding on their parent C atom.

### Crystal data

**Table:**

C_17_H_15_ClN_2_O_5_	*Z* = 2
*M**_r_* = 362.76	*F*(000) = 376
Triclinic, *P*1	*D*_x_ = 1.423 Mg m^−^^3^
Hall symbol: -P 1	Mo *K*α radiation, λ = 0.71073 Å
*a* = 8.3784 (3) Å	Cell parameters from 7904 reflections
*b* = 10.1071 (4) Å	θ = 2.5–27.5°
*c* = 10.7027 (4) Å	µ = 0.26 mm^−^^1^
α = 102.489 (2)°	*T* = 150 K
β = 103.977 (2)°	Block, colourless
γ = 96.026 (2)°	0.28 × 0.21 × 0.12 mm
*V* = 846.77 (6) Å^3^	

### Data collection

**Table:**

Bruker APEXII diffractometer	3369 reflections with *I* > 2σ(*I*)
graphite	*R*_int_ = 0.020
CCD rotation images, thin slices scans	θ_max_ = 27.5°, θ_min_ = 3.0°
Absorption correction: multi-scan (*SADABS*; Sheldrick, 2002)	*h* = −10→10
*T*_min_ = 0.842, *T*_max_ = 0.970	*k* = −13→13
12867 measured reflections	*l* = −13→10
3803 independent reflections	

### Refinement

**Table:**

Refinement on *F*^2^	Primary atom site location: structure-invariant direct methods
Least-squares matrix: full	Secondary atom site location: difference Fourier map
*R*[*F*^2^ > 2σ(*F*^2^)] = 0.049	Hydrogen site location: inferred from neighbouring sites
*wR*(*F*^2^) = 0.141	H-atom parameters constrained
*S* = 1.02	*w* = 1/[σ^2^(*F*_o_^2^) + (0.0775*P*)^2^ + 0.5515*P*] where *P* = (*F*_o_^2^ + 2*F*_c_^2^)/3
3803 reflections	(Δ/σ)_max_ = 0.006
229 parameters	Δρ_max_ = 0.54 e Å^−^^3^
0 restraints	Δρ_min_ = −0.42 e Å^−^^3^

### Special details

**Table:**

Geometry. All e.s.d.'s (except the e.s.d. in the dihedral angle between two l.s. planes) are estimated using the full covariance matrix. The cell e.s.d.'s are taken into account individually in the estimation of e.s.d.'s in distances, angles and torsion angles; correlations between e.s.d.'s in cell parameters are only used when they are defined by crystal symmetry. An approximate (isotropic) treatment of cell e.s.d.'s is used for estimating e.s.d.'s involving l.s. planes.
Refinement. Refinement of *F*^2^ against ALL reflections. The weighted *R*-factor *wR* and goodness of fit *S* are based on *F*^2^, conventional *R*-factors *R* are based on *F*, with *F* set to zero for negative *F*^2^. The threshold expression of *F*^2^ > σ(*F*^2^) is used only for calculating *R*-factors(gt) *etc*. and is not relevant to the choice of reflections for refinement. *R*-factors based on *F*^2^ are statistically about twice as large as those based on *F*, and *R*- factors based on ALL data will be even larger.

### Fractional atomic coordinates and isotropic or equivalent isotropic displacement parameters (Å^2^)

**Table:**

	*x*	*y*	*z*	*U*_iso_*/*U*_eq_	
Cl1	0.21857 (5)	−0.03042 (5)	1.03331 (5)	0.03620 (16)	
C1	0.3363 (2)	0.09251 (17)	0.98421 (17)	0.0277 (4)	
N2	0.45074 (18)	0.18016 (15)	1.07892 (14)	0.0279 (3)	
C3	0.5441 (2)	0.27800 (17)	1.04347 (17)	0.0274 (4)	
C4	0.6751 (2)	0.37174 (18)	1.14484 (18)	0.0312 (4)	
O5	0.69369 (17)	0.35382 (14)	1.27008 (13)	0.0379 (3)	
C6	0.8178 (3)	0.4523 (3)	1.3736 (2)	0.0508 (6)	
H6A	0.9272	0.448	1.3567	0.076*	
H6B	0.8202	0.4318	1.4593	0.076*	
H6C	0.7909	0.5447	1.3756	0.076*	
C7	0.7706 (2)	0.4691 (2)	1.1101 (2)	0.0371 (4)	
H7	0.8587	0.5309	1.177	0.045*	
C8	0.7410 (3)	0.4798 (2)	0.9768 (2)	0.0387 (4)	
H8	0.8091	0.5483	0.9558	0.046*	
C9	0.6153 (2)	0.3923 (2)	0.8783 (2)	0.0349 (4)	
O10	0.5731 (2)	0.39496 (16)	0.74704 (15)	0.0439 (4)	
C11	0.6705 (3)	0.4986 (2)	0.7119 (2)	0.0502 (6)	
H11A	0.6632	0.5894	0.7635	0.075*	
H11B	0.628	0.4926	0.6168	0.075*	
H11C	0.7871	0.4848	0.7313	0.075*	
C12	0.5154 (2)	0.28768 (18)	0.90951 (18)	0.0307 (4)	
C13	0.3894 (2)	0.18913 (19)	0.81199 (18)	0.0326 (4)	
H13	0.3669	0.1923	0.7214	0.039*	
C14	0.2996 (2)	0.08919 (18)	0.84700 (17)	0.0309 (4)	
C15	0.1747 (3)	−0.0235 (2)	0.74667 (18)	0.0367 (4)	
H15	0.1828	−0.1184	0.7581	0.044*	
O16	0.13334 (18)	−0.01119 (16)	0.61244 (13)	0.0421 (4)	
C17	0.0041 (2)	−0.00089 (19)	0.67835 (18)	0.0347 (4)	
C18	−0.0392 (2)	0.1364 (2)	0.7104 (2)	0.0379 (4)	
N19	−0.0698 (2)	0.2443 (2)	0.7373 (3)	0.0590 (6)	
C20	−0.1344 (3)	−0.1243 (2)	0.6298 (2)	0.0429 (5)	
O21	−0.1073 (3)	−0.23961 (17)	0.6035 (2)	0.0796 (7)	
O22	−0.27917 (19)	−0.08591 (15)	0.62274 (14)	0.0423 (4)	
C23	−0.4215 (3)	−0.1992 (3)	0.5871 (3)	0.0537 (6)	
H23A	−0.4089	−0.275	0.516	0.064*	
H23B	−0.4248	−0.2353	0.6655	0.064*	
C24	−0.5687 (4)	−0.1501 (3)	0.5431 (4)	0.0786 (10)	
H24A	−0.5758	−0.07	0.6108	0.118*	
H24B	−0.6643	−0.2222	0.5272	0.118*	
H24C	−0.5697	−0.1233	0.4602	0.118*	

### Atomic displacement parameters (Å^2^)

**Table:**

	*U*^11^	*U*^22^	*U*^33^	*U*^12^	*U*^13^	*U*^23^
Cl1	0.0300 (2)	0.0350 (3)	0.0367 (3)	−0.00048 (17)	0.00372 (17)	0.00336 (18)
C1	0.0245 (8)	0.0253 (8)	0.0276 (8)	0.0055 (6)	0.0001 (6)	0.0016 (6)
N2	0.0247 (7)	0.0284 (7)	0.0248 (7)	0.0059 (6)	−0.0002 (5)	0.0013 (6)
C3	0.0245 (8)	0.0254 (8)	0.0274 (8)	0.0075 (6)	0.0008 (6)	0.0018 (6)
C4	0.0277 (8)	0.0294 (9)	0.0292 (9)	0.0059 (7)	−0.0003 (7)	0.0009 (7)
O5	0.0337 (7)	0.0392 (7)	0.0265 (7)	−0.0048 (6)	−0.0059 (5)	−0.0005 (5)
C6	0.0442 (12)	0.0547 (13)	0.0318 (11)	−0.0119 (10)	−0.0056 (8)	−0.0069 (9)
C7	0.0311 (9)	0.0294 (9)	0.0416 (11)	0.0026 (7)	0.0007 (8)	0.0012 (8)
C8	0.0392 (10)	0.0312 (9)	0.0458 (11)	0.0059 (8)	0.0114 (9)	0.0098 (8)
C9	0.0392 (10)	0.0316 (9)	0.0350 (10)	0.0110 (8)	0.0096 (8)	0.0087 (8)
O10	0.0538 (9)	0.0443 (8)	0.0356 (8)	0.0093 (7)	0.0116 (6)	0.0144 (6)
C11	0.0678 (15)	0.0415 (12)	0.0497 (13)	0.0123 (11)	0.0237 (11)	0.0188 (10)
C12	0.0309 (9)	0.0293 (9)	0.0290 (9)	0.0109 (7)	0.0032 (7)	0.0040 (7)
C13	0.0379 (10)	0.0318 (9)	0.0235 (8)	0.0124 (7)	0.0005 (7)	0.0033 (7)
C14	0.0308 (9)	0.0287 (8)	0.0252 (8)	0.0093 (7)	−0.0029 (7)	−0.0004 (7)
C15	0.0411 (10)	0.0302 (9)	0.0272 (9)	0.0067 (8)	−0.0045 (7)	−0.0016 (7)
O16	0.0419 (8)	0.0488 (8)	0.0239 (7)	0.0100 (6)	−0.0022 (5)	−0.0037 (6)
C17	0.0367 (10)	0.0301 (9)	0.0269 (9)	0.0031 (7)	−0.0036 (7)	0.0003 (7)
C18	0.0265 (9)	0.0329 (10)	0.0426 (11)	−0.0005 (7)	−0.0027 (7)	0.0008 (8)
N19	0.0366 (10)	0.0347 (10)	0.0865 (16)	0.0036 (8)	−0.0016 (10)	−0.0033 (10)
C20	0.0435 (11)	0.0354 (10)	0.0332 (10)	−0.0011 (8)	−0.0092 (8)	−0.0004 (8)
O21	0.0689 (12)	0.0308 (9)	0.1015 (17)	0.0017 (8)	−0.0163 (11)	−0.0143 (9)
O22	0.0436 (8)	0.0386 (8)	0.0362 (7)	−0.0092 (6)	0.0032 (6)	0.0081 (6)
C23	0.0507 (13)	0.0498 (13)	0.0491 (13)	−0.0164 (10)	0.0050 (10)	0.0094 (10)
C24	0.0601 (17)	0.0529 (16)	0.126 (3)	0.0022 (13)	0.0284 (18)	0.0286 (18)

### Geometric parameters (Å, °)

**Table:**

Cl1—C1	1.7514 (19)	C11—H11C	0.98
C1—N2	1.302 (2)	C12—C13	1.412 (3)
C1—C14	1.418 (2)	C13—C14	1.366 (3)
N2—C3	1.370 (2)	C13—H13	0.95
C3—C12	1.422 (2)	C14—C15	1.492 (2)
C3—C4	1.429 (2)	C15—O16	1.430 (2)
C4—O5	1.365 (2)	C15—C17	1.505 (3)
C4—C7	1.372 (3)	C15—H15	1
O5—C6	1.431 (2)	O16—C17	1.429 (3)
C6—H6A	0.98	C17—C18	1.461 (3)
C6—H6B	0.98	C17—C20	1.516 (3)
C6—H6C	0.98	C18—N19	1.139 (3)
C7—C8	1.417 (3)	C20—O21	1.197 (3)
C7—H7	0.95	C20—O22	1.302 (3)
C8—C9	1.367 (3)	O22—C23	1.478 (3)
C8—H8	0.95	C23—C24	1.393 (4)
C9—O10	1.371 (2)	C23—H23A	0.99
C9—C12	1.425 (3)	C23—H23B	0.99
O10—C11	1.430 (3)	C24—H24A	0.98
C11—H11A	0.98	C24—H24B	0.98
C11—H11B	0.98	C24—H24C	0.98
			
N2—C1—C14	125.66 (17)	C14—C13—C12	120.43 (17)
N2—C1—Cl1	116.16 (14)	C14—C13—H13	119.8
C14—C1—Cl1	118.18 (13)	C12—C13—H13	119.8
C1—N2—C3	117.43 (15)	C13—C14—C1	116.95 (16)
N2—C3—C12	122.01 (15)	C13—C14—C15	122.41 (17)
N2—C3—C4	118.49 (16)	C1—C14—C15	120.56 (17)
C12—C3—C4	119.50 (17)	O16—C15—C14	116.67 (17)
O5—C4—C7	125.88 (16)	O16—C15—C17	58.19 (12)
O5—C4—C3	115.19 (16)	C14—C15—C17	122.08 (16)
C7—C4—C3	118.92 (17)	O16—C15—H15	115.8
C4—O5—C6	115.66 (16)	C14—C15—H15	115.8
O5—C6—H6A	109.5	C17—C15—H15	115.8
O5—C6—H6B	109.5	C17—O16—C15	63.54 (12)
H6A—C6—H6B	109.5	O16—C17—C18	114.72 (17)
O5—C6—H6C	109.5	O16—C17—C15	58.27 (12)
H6A—C6—H6C	109.5	C18—C17—C15	118.78 (16)
H6B—C6—H6C	109.5	O16—C17—C20	114.39 (16)
C4—C7—C8	121.65 (18)	C18—C17—C20	118.84 (17)
C4—C7—H7	119.2	C15—C17—C20	117.05 (17)
C8—C7—H7	119.2	N19—C18—C17	178.6 (2)
C9—C8—C7	120.54 (19)	O21—C20—O22	127.0 (2)
C9—C8—H8	119.7	O21—C20—C17	122.2 (2)
C7—C8—H8	119.7	O22—C20—C17	110.79 (17)
C8—C9—O10	125.59 (18)	C20—O22—C23	115.01 (18)
C8—C9—C12	119.68 (18)	C24—C23—O22	109.0 (2)
O10—C9—C12	114.73 (17)	C24—C23—H23A	109.9
C9—O10—C11	116.36 (18)	O22—C23—H23A	109.9
O10—C11—H11A	109.5	C24—C23—H23B	109.9
O10—C11—H11B	109.5	O22—C23—H23B	109.9
H11A—C11—H11B	109.5	H23A—C23—H23B	108.3
O10—C11—H11C	109.5	C23—C24—H24A	109.5
H11A—C11—H11C	109.5	C23—C24—H24B	109.5
H11B—C11—H11C	109.5	H24A—C24—H24B	109.5
C13—C12—C3	117.46 (17)	C23—C24—H24C	109.5
C13—C12—C9	122.83 (17)	H24A—C24—H24C	109.5
C3—C12—C9	119.69 (17)	H24B—C24—H24C	109.5
			
C14—C1—N2—C3	0.1 (3)	C12—C13—C14—C1	1.7 (3)
Cl1—C1—N2—C3	−179.64 (12)	C12—C13—C14—C15	−175.11 (16)
C1—N2—C3—C12	1.9 (2)	N2—C1—C14—C13	−1.9 (3)
C1—N2—C3—C4	−177.41 (15)	Cl1—C1—C14—C13	177.79 (13)
N2—C3—C4—O5	−0.2 (2)	N2—C1—C14—C15	174.96 (17)
C12—C3—C4—O5	−179.61 (15)	Cl1—C1—C14—C15	−5.3 (2)
N2—C3—C4—C7	179.25 (16)	C13—C14—C15—O16	−9.4 (3)
C12—C3—C4—C7	−0.1 (3)	C1—C14—C15—O16	173.87 (16)
C7—C4—O5—C6	4.2 (3)	C13—C14—C15—C17	−77.0 (3)
C3—C4—O5—C6	−176.33 (17)	C1—C14—C15—C17	106.3 (2)
O5—C4—C7—C8	−179.77 (17)	C14—C15—O16—C17	−112.82 (19)
C3—C4—C7—C8	0.8 (3)	C15—O16—C17—C18	109.79 (18)
C4—C7—C8—C9	−0.1 (3)	C15—O16—C17—C20	−107.86 (18)
C7—C8—C9—O10	178.50 (18)	C14—C15—C17—O16	103.6 (2)
C7—C8—C9—C12	−1.3 (3)	O16—C15—C17—C18	−102.8 (2)
C8—C9—O10—C11	0.2 (3)	C14—C15—C17—C18	0.8 (3)
C12—C9—O10—C11	179.96 (17)	O16—C15—C17—C20	103.26 (19)
N2—C3—C12—C13	−2.0 (2)	C14—C15—C17—C20	−153.18 (19)
C4—C3—C12—C13	177.30 (15)	O16—C17—C20—O21	40.3 (3)
N2—C3—C12—C9	179.44 (15)	C18—C17—C20—O21	−179.0 (2)
C4—C3—C12—C9	−1.2 (3)	C15—C17—C20—O21	−25.1 (3)
C8—C9—C12—C13	−176.53 (17)	O16—C17—C20—O22	−140.08 (17)
O10—C9—C12—C13	3.7 (3)	C18—C17—C20—O22	0.6 (3)
C8—C9—C12—C3	1.9 (3)	C15—C17—C20—O22	154.55 (18)
O10—C9—C12—C3	−177.88 (15)	O21—C20—O22—C23	4.5 (4)
C3—C12—C13—C14	0.1 (3)	C17—C20—O22—C23	−175.12 (17)
C9—C12—C13—C14	178.57 (16)	C20—O22—C23—C24	−161.8 (2)

### Hydrogen-bond geometry (Å, °)

**Table:**

*D*—H···*A*	*D*—H	H···*A*	*D*···*A*	*D*—H···*A*
C7—H7···N19^i^	0.95	2.57	3.434 (3)	151
C24—H24A···O16^ii^	0.98	2.57	3.152 (4)	118
C13—H13···O16	0.95	2.54	2.875 (2)	101

Symmetry codes: (i) −*x*+1, −*y*+1, −*z*+2; (ii) *x*−1, *y*, *z*.
